# Differences in Clinical Presentation With Long COVID After Community and Hospital Infection and Associations With All-Cause Mortality: English Sentinel Network Database Study

**DOI:** 10.2196/37668

**Published:** 2022-08-16

**Authors:** Bernardo Meza-Torres, Gayathri Delanerolle, Cecilia Okusi, Nikhil Mayor, Sneha Anand, Jack Macartney, Piers Gatenby, Ben Glampson, Martin Chapman, Vasa Curcin, Erik Mayer, Mark Joy, Trisha Greenhalgh, Brendan Delaney, Simon de Lusignan

**Affiliations:** 1 Nuffield Department of Primary Care Health Sciences University of Oxford Oxford United Kingdom; 2 Royal Surrey NHS Foundation Trust Guildford United Kingdom; 3 Imperial College Healthcare NHS Trust Imperial Clinical Analytics, Research & Evaluation (iCARE) London United Kingdom; 4 King's College London Population Health Sciences London United Kingdom; 5 Department of Surgery & Cancer Institute of Global Health Innovation Imperial College London London United Kingdom

**Keywords:** medical record systems, computerized, Systematized Nomenclature of Medicine, post–acute COVID-19 syndrome, phenotype, COVID-19, post–COVID-19 syndrome, long COVID, ethnicity, social class, general practitioners, data accuracy, data extracts, biomedical ontologies, SARS-CoV-2, hospitalization

## Abstract

**Background:**

Most studies of long COVID (symptoms of COVID-19 infection beyond 4 weeks) have focused on people hospitalized in their initial illness. Long COVID is thought to be underrecorded in UK primary care electronic records.

**Objective:**

We sought to determine which symptoms people present to primary care after COVID-19 infection and whether presentation differs in people who were not hospitalized, as well as post–long COVID mortality rates.

**Methods:**

We used routine data from the nationally representative primary care sentinel cohort of the Oxford–Royal College of General Practitioners Research and Surveillance Centre (N=7,396,702), applying a predefined long COVID phenotype and grouped by whether the index infection occurred in hospital or in the community. We included COVID-19 infection cases from March 1, 2020, to April 1, 2021. We conducted a before-and-after analysis of long COVID symptoms prespecified by the Office of National Statistics, comparing symptoms presented between 1 and 6 months after the index infection matched with the same months 1 year previously. We conducted logistic regression analysis, quoting odds ratios (ORs) with 95% CIs.

**Results:**

In total, 5.63% (416,505/7,396,702) and 1.83% (7623/416,505) of the patients had received a coded diagnosis of COVID-19 infection and diagnosis of, or referral for, long COVID, respectively. People with diagnosis or referral of long COVID had higher odds of presenting the prespecified symptoms after versus before COVID-19 infection (OR 2.66, 95% CI 2.46-2.88, for those with index community infection and OR 2.42, 95% CI 2.03-2.89, for those hospitalized). After an index community infection, patients were more likely to present with nonspecific symptoms (OR 3.44, 95% CI 3.00-3.95; *P*<.001) compared with after a hospital admission (OR 2.09, 95% CI 1.56-2.80; *P*<.001). Mental health sequelae were more strongly associated with index hospital infections (OR 2.21, 95% CI 1.64-2.96) than with index community infections (OR 1.36, 95% CI 1.21-1.53; *P*<.001). People presenting to primary care after hospital infection were more likely to be men (OR 1.43, 95% CI 1.25-1.64; *P*<.001), more socioeconomically deprived (OR 1.42, 95% CI 1.24-1.63; *P*<.001), and with higher multimorbidity scores (OR 1.41, 95% CI 1.26-1.57; *P*<.001) than those presenting after an index community infection. All-cause mortality in people with long COVID was associated with increasing age, male sex (OR 3.32, 95% CI 1.34-9.24; *P*=.01), and higher multimorbidity score (OR 2.11, 95% CI 1.34-3.29; *P*<.001). Vaccination was associated with reduced odds of mortality (OR 0.10, 95% CI 0.03-0.35; *P*<.001).

**Conclusions:**

The low percentage of people recorded as having long COVID after COVID-19 infection reflects either low prevalence or underrecording. The characteristics and comorbidities of those presenting with long COVID after a community infection are different from those hospitalized. This study provides insights into the presentation of long COVID in primary care and implications for workload.

## Introduction

### Background

Long COVID (LC) is defined as fatigue, breathlessness, cognitive dysfunction, and a variety of other symptoms occurring after COVID-19 infection [1,2]. More than 1 million people in the United Kingdom are estimated to have prolonged symptoms after COVID-19 infection, with 60% of the patients with long COVID reporting extended symptoms lasting months and 240,000 people reporting symptoms that limit day-to-day activity [3,4]. The spectrum of symptoms implies widespread involvement of organs, and there is a recognizable pattern of long COVID disease resulting from autonomic dysfunction and mast cell disorder [5]. The Office for National Statistics (ONS) suggests that the prevalence of long COVID is greater in women, middle-aged people, those from the most deprived areas, and those with an activity-limiting health condition or disability [4]. Symptoms are wide ranging, but fatigue, shortness of breath, and cognitive difficulties (termed *brain fog* by patients) are most commonly reported [6-8]. In late 2020, there was a release of International Classification of Disease and Systematized Nomenclature of Medicine Clinical Terms (SNOMED CT) to support long COVID coding (termed post–COVID-19 condition) but recording in primary care electronic records varied [9,10]. However, primary care data remain the most useful source of epidemiological data outside hospital records and bespoke surveys to understand the symptoms that patients with long COVID present to primary care after documented COVID-19 infection [6]. There is a need to characterize the prevalence, risk factors, and symptom patterns in patients with long COVID using routine clinical data to understand the symptoms that people present with at primary care facilities after COVID-19 infection and whether presentation and postacute mortality differ in people who were not hospitalized.

### This Study

This study reports the symptoms, sociodemographic profile, and outcomes of people identified as having long COVID in English primary care. Our study has four components: (1) a comparison of clinical symptoms of people with long COVID before and after COVID-19 infection, (2) a description of the characteristics of people with long COVID compared with those without long COVID, (3) a comparison of those with long COVID who were hospitalized with COVID-19 infection versus those who were not, and (4) an analysis of all-cause mortality in people with long COVID.

## Methods

### Overview

This study was conducted as part of the Predicting Risk of Hospital Admission in Patients With Suspected COVID-19 in a Community Setting (Remote COVID-19 Assessment in Primary Care) project [11-13]. The project included creating a phenotype for LC through an observational study. The population characteristics, baseline data, and our LC phenotype were published in the study protocol [14]. The protocol also set out the details of the comparisons undertaken in this study. These were as follows: (1) undertaking a before-and-after comparison of the number of symptoms identified by the ONS as more common in LC; (2) comparing sociodemographic, comorbid, and exposure characteristics of people who had received a coded diagnosis of LC from their general practitioner (GP) with those of people who had not; (3) comparing characteristics of people with LC who had contracted their index infection in hospital with those of people who had contracted a community infection; and (4) an analysis of all-cause mortality in people with LC. The study period included COVID-19 infection cases between March 1, 2020, and April 1, 2021, with a follow-up period of a further 6 months, up to September 30, 2021.

### Study Population

**Figure 1:**
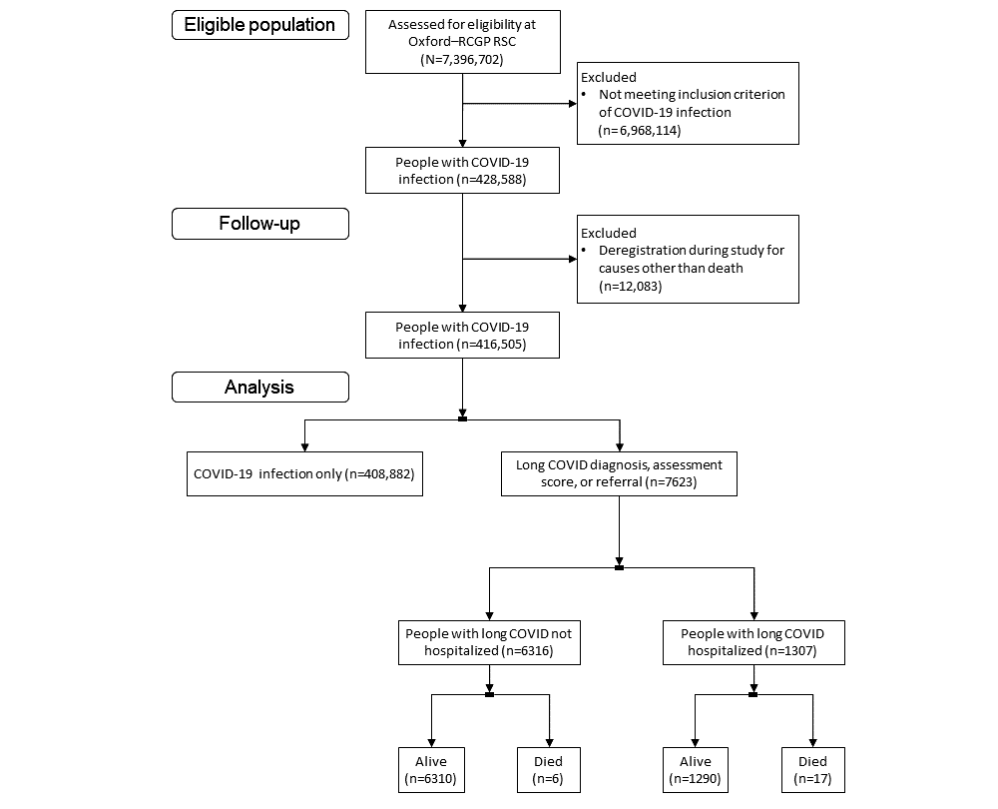
CONSORT (Consolidated Standards of Reporting Trials) diagram of the English primary care sentinel cohort population, the people with COVID-19 infection, those with long COVID, and the numbers of people with index community infection and those hospitalized for treatment for their index infection. Oxford–RCGP RSC: Oxford–Royal College of General Practitioners Research and Surveillance Centre.

We used pseudonymized data extracted from the primary care sentinel cohort (PCSC) of the Oxford–Royal College of General Practitioners Research and Surveillance Centre [15]. The PCSC includes 743 practices (N=7,396,702) that were recruited to be nationally representative of the English population, and it is one of Europe’s oldest sentinel systems [11]. PCSC data have been widely used in COVID-19 research [16]. Practices are encouraged to have high-quality records and to record cases of LC [10]. Key diagnoses in primary care in England are recorded in computerized medical records (CMRs) using SNOMED CT [17]. This includes COVID-19 test results and vaccination. Over the period of the study, all community COVID-19 test and vaccination data were posted electronically back into patients’ CMRs. We have previously found that 7.81% (58/743) of the practices did not have any LC cases recorded in their CMR systems, and these practices were excluded from the study. The registered population of the PCSC was 7,396,702 patients at the time of the study; after exclusions, approximately 6.9 million patients were included, and 6.15% (428,588/6,968,114) had COVID-19 infection recorded in their CMR (Figure 1).

### Long COVID Cases

LC cases were defined using our phenotype, with LC cases being defined by a clinical term for a case, referral to an LC service, or a symptom score suggestive of LC based on the ONS set of possible LC symptoms. The phenotype also differentiated community cases from hospital cases. Using this phenotype, 1.83% (7623/416,505) of the population who had been exposed to COVID-19 infection were recorded in the GP CMR as having LC; 82.85% (6316/7623) were index community COVID-19 infection cases, and 17.15% (1307/7623) had been hospitalized for treatment for their primary COVID-19 infection; and 0.3% (23/7623) of the people with a prior record of LC diagnosis had died. Most (7347/7623, 96.38%) of our LC cases had not received a vaccination dose before their diagnosis, 3.49% (266/7623) had received a single vaccine dose, and 0.13% (10/7623) had received 2 doses. We have set out how the PCSC population was subdivided to identify people with LC in Figure 1. Sociodemographic details included age, sex, use of the Index of Multiple Deprivation as a measure of deprivation, ethnicity, population density, obesity, and smoking. The Index of Multiple Deprivation score was dichotomized into the top 3 (*least*) and bottom 2 (*most*) deprived quintiles. Ethnicity was also dichotomized into White and non-White. Population density categories were based on the ONS national figures and categorized into conurbation (highest density), town and city (medium density), and rural (lowest density). Obesity was reported based on BMI>30 kg/m^2^ or a term for obesity from SNOMED CT. Symptoms associated with LC were those present >28 days after the index infection.

### Before-and-After Study

We compared the recording of symptoms associated with LC in the period between 1 and 6 months after the index COVID-19 infection. We made a historical comparison matched by month in the year before the index COVID-19 infection. We did this to make allowance for seasonality in symptom presentations in primary care.

Reporting frequencies and underreporting are likely to be similar to unrecorded cases of patients with LC. The comparisons between before-and-after symptoms reported among patients with LC before the pandemic were matched by month, provided that an acute COVID-19 infection code was present; for example, patients with a COVID-19 infection code entered on January 1, 2021, would have a follow-up period beginning from February 1, 2021, to July 31, 2021, with a historic comparator period from February 1, 2019, to July 31, 2019. This comparative method demonstrated the clinical phenotype variation.

### Comparing LC and Uncomplicated COVID-19 Infection

We compared people with LC with those with COVID-19 infection uncomplicated by subsequent LC. We made this comparison across all variables of interest, identified through a literature review reported in our protocol [14]. In addition, we included the Cambridge Multimorbidity Score (CMS) as an overall measure of comorbidity; although CMS was initially developed using the Read clinical terminology, we have subsequently validated it using SNOMED CT [17]. An increasing CMS is associated with greater levels of comorbidity and associated with increased risk of mortality [18]. We conducted a multivariate logistic regression analysis to characterize people with LC.

### Comparison of Hospitalized and Community Initial Infections

We used the same variables to make comparisons between patients who were hospitalized with their index COVID-19 infection and those who were not. We compared people with posthospitalization LC with those who had index community COVID-19 infection followed by LC.

### All-Cause Mortality as an Outcome for LC

We measured all-cause mortality as an outcome for all patients with LC drawn from the hospitalized and community groups. We conducted a multivariate analysis using age, sex, geographical location, CMS, and whether the patients had received their vaccination doses before or after their COVID-19 infection diagnosis.

### Statistical Methods

We conducted this secondary analysis of routine data from the PCSC, and full details are available in our protocol [18]. We reported descriptive statistics of mean, median, and proportion, with measures of dispersion such as SD and IQR for normally distributed data and nonparametric data, respectively. We conducted a univariate analysis reporting odds ratios (ORs) and 95% CIs. We used the Bonferroni correction to correct for multiple testing in our before-and-after study of symptoms associated with LC. The probability of an observed difference happening by chance (*P* values) were reported for categorical variables using chi-square tests. ANOVA was used for continuous variables.

Multivariate logistic regression modelling was used to identify comorbidities, demographics, and exposure covariates associated with (1) LC diagnosis, (2) hospitalization, and (3) all-cause mortality as binary outcomes in separate models. For each model, relevant risk factors identified in the literature underwent univariate analysis, and all covariates were then included in a 3-step backward elimination using thresholds of α levels of .20, .10, and .05 in each step respectively, where a 2-sided α value of .05 was considered statistically significant. Age and sex were forced into the model at each step. Results were presented through forest plots.

### Ethics Approval

Retrospective pseudonymized routine data were used for this study. These data are held at the Oxford–Royal College of General Practitioners Clinical Informatics Digital Hub, a trusted research environment [19] that meets the NHS Digital Data Security and Protection standards [20]. Ethics approval was granted by the North West–Greater Manchester East Research Ethics Committee and Health Research Authority on May 27, 2021 (Integrated Research Application System number: 283024; Research Ethics Committee reference number: 20/NW/0266).

## Results

### Cohort Summary

A total of 416,505 people had a record of acute COVID-19 infection between March 1, 2020, and April 1, 2021. Baseline characteristics of the population are reported in Table 1. The mean age of the patients was 44.5 (SD 21.7) years, with a majority being women (232,775/416,505, 55.89%). The most common comorbid conditions were obesity, anxiety, depression, eczema, hypertension, and asthma. The all-cause mortality rate within the study population was 4.08% (16,993/416,505). Only 1.81% (7531/416,505) of the deaths were attributable to complications related to COVID-19 infection.

**Table 1:** Frequencies of baseline characteristics and univariate odds ratios for people with COVID-19 infection stratified by long COVID status in the primary care sentinel cohort in England (March 1, 2020, to April 1, 2021; N=416,505).

Variable and category						COVID-19 infection, n=408,882			Long COVID, n=7623		Unadjusted odds ratio (95% CI)		*P* value	
**Sociodemographic characteristics**														
	**Age (years), mean (SD)**													
				Continuous		44.5 (21.77)			47.7 (14.82)		1.01 (1.01-1.01)		<.001	
	**Sex, n (%)**													
				Female (reference)		227,849 (55.7)			4926 (64.6)		1.00 (N/A^a^)		N/A	
				Male		181,033 (44.3)			2697 (35.4)		0.69 (0.66-0.72)		<.001	
	**Deprivation, n (%)**													
				Least deprived (reference)		164,001 (40.1)			3048 (40)		1.00 (N/A)		N/A	
				Most deprived		244,881 (59.9)			4575 (60)		1.01 (0.96-1.05)		.83	
	**Ethnicity, n (%)**													
				White (reference)		268,624 (65.7)			5529 (72.5)		1.00 (N/A)		N/A	
				Non-White		56,645 (13.9)			1094 (14.4)		0.94 (0.88-1.00)		.06	
				Missing		83,613 (20.4)			1000 (13.1)		0.58 (0.54-0.62)		<.001	
	**Population density, n (%)**													
				City (reference)		205,159 (50.2)			3191 (41.9)		1.00 (N/A)		N/A	
				Conurbation		137,378 (33.6)			3196 (41.9)		1.50 (1.42-1.57)		<.001	
				Rural		66,345 (16.2)			1236 (16.2)		1.20 (1.12-1.28)		<.001	
	**BMI, n (%)**													
				Nonobese (reference)		252,114 (61.7)			4522 (59.3)		1.00 (N/A)		N/A	
				Obese		101,386 (24.8)			2575 (33.8)		1.42 (1.35-1.49)		<.001	
				Missing		55,382 (13.5)			526 (6.9)		0.53 (0.48-0.58)		<.001	
	**Smoker, n (%)**													
				Nonsmoker (reference)		210,505 (51.5)			4458 (58.5)		1.00 (N/A)		N/A	
				Smoker or former smoker		149,583 (36.6)			2945 (38.6)		0.93 (0.89-0.97)		<.001	
				Missing		48,794 (11.9)			220 (2.9)		0.21 (0.19-0.24)		<.001	
**Comorbidities**														
	**Depression, n (%)**													
				No (reference)		315,510 (77.2)			4862 (63.8)		1.00 (N/A)		N/A	
				Yes		93,372 (22.8)			2761 (36.2)		1.92 (1.83-2.01)		<.001	
	**Anxiety, n (%)**													
				No (reference)		313,782 (76.7)			4969 (65.2)		1.00 (N/A)		N/A	
				Yes		95,100 (23.3)			2654 (34.8)		1.76 (1.68-1.85)		<.001	
	**Asthma, n (%)**													
				No (reference)		333,083 (81.5)			5821 (76.4)		1.00 (N/A)		N/A	
				Yes		75,799 (18.5)			1802 (23.6)		1.36 (1.29-1.43)		<.001	
	**Chronic lung disease, n (%)**													
				No (reference)		394,414 (96.5)			7429 (97.5)		1.00 (N/A)		N/A	
				Yes		14,468 (3.5)			194 (2.5)		0.71 (0.62-0.82)		<.001	
	**COPD^b^, n (%)**													
			No (reference)		396,024 (96.9)			7473 (98)		1.00 (N/A)		N/A		
			Yes		12,858 (3.1)			150 (2)		0.62 (0.53-0.73)		<.001		
		**Hypertension, n (%)**												
			No (reference)		328,025 (80.2)			6001 (78.7)		1.00 (N/A)		N/A		
			Yes		80,857 (19.8)			1622 (21.3)		1.10 (1.04-1.16)		<.001		
		**Ischemic heart disease**												
			No (reference)		387,015 (94.7)			7283 (95.5)		1.00 (N/A)		N/A		
			Yes		21,867 (5.3)			340 (4.5)		0.83 (0.74-0.92)		<.001		
		**Atrial fibrillation, n (%)**												
			No (reference)		395,170 (96.6)			7490 (98.3)		1.00 (N/A)		N/A		
			Yes		13,712 (3.4)			133 (1.7)		0.51 (0.43-0.61)		<.001		
		**Congestive heart failure, n (%)**												
			No (reference)		400,573 (98)			7558 (99.1)		1.00 (N/A)		N/A		
			Yes		8309 (2)			65 (0.9)		0.41 (0.32-0.53)		<.001		
		**CKD^c^, n (%)**												
			No (reference)		385,985 (94.4)			7350 (96.4)		1.00 (N/A)		N/A		
			Yes		22,897 (5.6)			273 (3.6)		0.63 (0.55-0.71)		<.001		
		**Type 2 diabetes, n (%)**												
			No (reference)		378,258 (92.5)			7042 (92.4)		1.00 (N/A)		N/A		
			Yes		30,624 (7.5)			581 (7.6)		1.02 (0.94-1.11)		.67		
		**Type 1 diabetes, n (%)**												
			No (reference)		406,311 (99.4)			7581 (99.4)		1.00 (N/A)		N/A		
			Yes		2571 (0.6)			42 (0.6)		0.88 (0.64-1.19)		.38		
		**Cirrhosis, n (%)**												
			No (reference)		407,827 (99.7)			7607 (99.8)		1.00 (N/A)		N/A		
			Yes		1055 (0.3)			16 (0.2)		0.81 (0.50-1.33)		.40		
		**Eczema, n (%)**												
			No (reference)		318,124 (77.8)			5891 (77.3)		1.00 (N/A)		N/A		
			Yes		90,758 (22.2)			1732 (22.7)		1.03 (0.98-1.09)		.28		
		**CMS^d^, mean (SD)**												
			Continuous		0.45 (1.59)			0.29 (1.12)		0.94 (0.92-0.95)		<.001		
**Exposures**														
		**ICU^e^ admission, n (%) **												
			No (reference)		406,302 (99.4)			7351 (96.4)		1.00 (N/A)		N/A		
			Yes		2580 (0.6)			272 (3.6)		5.83 (5.13-6.62)		<.001		
		**Vaccination at any time, n (%)**												
			No vaccine (reference)		84,094 (20.6)			872 (11.4)		1.00 (N/A)		N/A		
			One dose		25,571 (6.3)			371 (4.9)		1.40 (1.24-1.58)		<.001		
			Two doses		299,217 (73.2)			6380 (83.7)		2.06 (1.92-2.21)		<.001		
		**Pre–** **long COVID** **vaccination, n (%)**												
			No vaccine (reference)		392,324 (96)			7347 (96.4)		1.00 (N/A)		N/A		
			One dose		15,832 (3.9)			266 (3.5)		0.90 (0.79-1.01)		.08		
			Two doses		726 (0.2)			10 (0.1)		0.74 (0.39-1.37)		.31		
**Outcomes**														
		**All-cause mortality, n (%)**												
			No (reference)		391,912 (95.8)			7600 (99.7)		1.00 (N/A)		N/A		
			Yes		16,970 (4.2)			23 (0.3)		0.07 (0.05-0.11)		<.001		

^a^N/A: not applicable.

^b^COPD: chronic obstructive pulmonary disease.

^c^CKD: chronic kidney disease.

^d^CMS: Cambridge Multimorbidity Score.

^e^ICU: intensive care unit.

### Before-and-After Study

**Figure 2:**
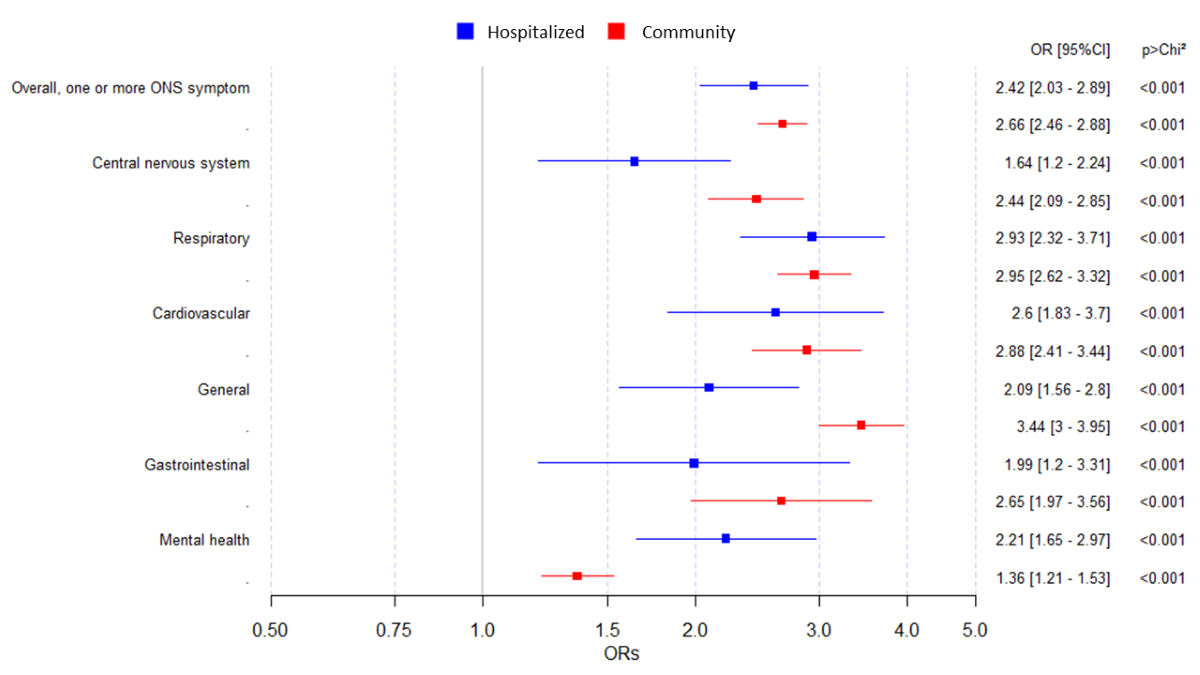
Change in long COVID symptom presentation by symptom category for people who contracted a posthospitalization and index community COVID-19 infection. Univariate odds ratios (ORs) and 95% CIs are presented for COVID-19 infection cases in the primary care sentinel cohort in England between March 1, 2020, and April 1, 2021. ONS: Office for National Statistics.

Overall, symptomatic presentations to primary care increased in people after their diagnosis compared with a matched historic period. The odds of presenting with these symptoms more than doubled. The increased ORs were 2.66 (95% CI 2.46-2.88) and 2.42 (95% CI 2.03-2.89) for community and hospitalized patients, respectively (Figure 2).

There were no differences between people who had been hospitalized with COVID-19 infection and those who had contracted community infections by category, other than the differences in general and mental health symptoms. Patients presented with more general symptoms after an index community infection (OR 3.44, 95% CI 3.00-3.95) than after an index hospital infection (OR 2.09, 95% CI 1.56-2.80; *P*<.001). Presentations with mental health sequelae were associated more with index hospital infections (OR 2.21, 95% CI 1.64-2.96) than with index community infections (OR 1.36, 95% CI 1.21-1.53).

There was an overall increase in reporting individual symptoms for 95% (20/21) of the symptoms monitored in both the index hospital and community infection groups. Among those hospitalized, shortness of breath (OR 15.8, 95% CI 9.5-26.4), loss of taste (OR 6.0, 95% CI 0.73-50.0), and memory loss and confusion (OR 5.0, 95% CI 0.58-43.32) were the symptoms that showed a higher increase after LC. For the community group, difficulty concentrating (OR 11.7, 95% CI 3.6-38.0), loss of taste (OR 8.7, 95% CI 3.4-21.7), and loss of smell (OR 7.5, 95% CI 4.2-13.2) showed a higher increase after LC. Only abdominal pain in the hospitalized group saw a decrease after LC versus before LC (Table 2).

**Table 2:** Change in symptoms in the hospitalized and community groups before and after developing long COVID for people presenting with COVID-19 infection in the primary care sentinel cohort in England between March 1, 2020, and April 1, 2021 (N=7609).

ONS^a^ symptom variables			Long COVID: hospitalized (n=1294)							Unadjusted OR^b^ (95% CI)			*P* value			Long COVID: community (n=6315)							Unadjusted OR (95% CI)			*P* value
			Before, n (%)		After, n (%)		Difference, %								Before, n (%)			After, n (%)		Difference, %						
Overall, one or more ONS symptom^c^			315 (24.34)		567 (43.82)		19.48		2.42 (2.03-2.89)			<.001			1326 (21)			2616 (41.43)		20.43		2.66 (2.46-2.88)			<.001	
**Central nervous system^c^**			65 (5.02)		103 (7.96)		2.94		1.64 (1.2-2.24)			.004			250 (3.96)			578 (9.15)		5.19		2.44 (2.09-2.86)			<.001	
	Memory loss and confusion	1 (0.08)		5 (0.39)		0.31		5.02 (0.58-43.32)			.22			4 (0.06)			14 (0.22)		0.16		3.51 (1.15-10.71)			.03		
	Difficulty concentrating	0		6 (0.46)		0.46		Inf^d^			.04			3 (0.23)			35 (0.55)		0.32		11.73 (3.62-38.01)			<.001		
	Loss of smell	3 (0.23)		6 (0.46)		0.23		2 (0.5-8.06)			.50			14 (0.22)			103 (1.63)		1.41		7.46 (4.23-13.17)			<.001		
	Trouble sleeping	9 (0.7)		21 (1.62)		0.92		2.36 (1.08-5.16)			.05			19 (0.3)			46 (0.73)		0.43		2.43 (1.43-4.13)			<.001		
	Headache	37 (2.86)		48 (3.71)		0.85		1.31 (0.85-2.01)			.28			158 (2.5)			306 (4.85)		2.35		1.98 (1.63-2.41)			<.001		
	Loss of taste	1 (0.08)		6 (0.46)		0.38		6.02 (0.73-50.02)			.12			5 (0.08)			43 (0.68)		0.6		8.65 (3.44-21.74)			<.001		
	Vertigo and dizziness	16 (1.24)		30 (2.32)		1.08		1.9 (1.03-3.48)			.05			66 (1)			137 (2.17)		1.17		2.1 (1.56-2.82)			<.001		
**Respiratory^c^**			118 (9.12)		294 (22.72)		13.6		2.93 (2.32-3.71)			<.001			416 (6.59)			1088 (17.24)		10.65		2.95 (2.62-3.32)			<.001	
	Sore throat	16 (1.24)		18 (1.39)		0.15		0.36 (0.21-0.62)			.86			83 (1.31)			77 (1.22)		–0.09		0.93 (0.68-1.27)			.71		
	Shortness of breath	49 (3.79)		214 (16.54)		12.75		15.83 (9.51-26.35)			<.001			150 (2.38)			714 (11.31)		8.93		5.24 (4.39-6.25)			<.001		
	Cough	70 (5.41)		114 (8.81)		3.4		1.69 (1.23-2.31)			.002			250 (3.96)			544 (8.61)		4.65		2.29 (1.96-2.68)			<.001		
**Cardiovascular^c^**			46 (3.55)		113 (8.73)		5.18		2.6 (1.82-3.69)			<.001			171 (2.71)			468 (7.41)		4.7		2.88 (2.41-3.43)			<.001	
	Palpitations	9 (0.7)		26 (2)		1.3		2.93 (1.36-6.29)			.007			38 (0.6)			128 (2.01)		1.41		3.42 (2.35-4.96)			<.001		
	Chest pain	38 (2.94)		97 (7.5)		4.6		2.68 (1.81-3.96)			.001			143 (2.26)			371 (5.87)		3.61		2.69 (2.21-3.28)			<.001		
**General^c^**			78 (6.03)		153 (11.82)		5.79		2.09 (1.56-2.8)			<.001			341 (5.4)			1037 (16.42)		11.02		3.44 (3-3.95)			<.001	
	Weakness and tiredness	26 (2.01)		95 (7.34)		5.33		3.86 (2.51-5.95)			.001			123 (1.95)			786 (12.45)		10.5		7.16 (5.88-8.71)			<.001		
	Fever	11 (0.85)		20 (1.55)		0.7		1.83 (0.87-3.86)			.16			48 (0.76)			105 (1.66)		0.9		2.21 (1.55-3.14)			<.001		
	Muscle aches	8 (0.62)		24 (1.85)		1.23		3.04 (1.36-6.79)			.004			33 (0.52)			121 (1.92)		1.4		3.72 (2.51-5.5)			<.001		
	Abdominal pain	40 (3.09)		37 (2.86)		–0.23		0.92 (0.59-1.45)			.83			158 (2.5)			178 (2.82)		0.32		1.13 (0.91-1.4)			.30		
**Gastrointestinal^c^**			23 (1.78)		45 (3.48)		1.7		1.99 (1.2-3.31)			.01			64 (1.01)			167 (2.64)		1.63		2.65 (1.98-3.56)			<.001	
	Nausea and vomiting	7 (0.54)		23 (1.78)		1.24		3.33 (1.43-7.73)			.006			27 (0.43)			72 (1.14)		0.71		2.69 (1.71-4.22)			<.001		
	Loss of appetite	2 (0.15)		6 (0.46)		0.31		3.01 (0.6-15.01)			.29			9 (0.14)			35 (0.55)		0.41		3.9 (1.89-8.06)			<.001		
	Diarrhea	16 (1.24)		25 (1.93)		0.69		1.57 (0.84-2.95)			.21			34 (0.54)			79 (1.25)		0.71		2.34 (1.55-3.53)			<.001		
**Mental health^c^**			70 (5.41)		145 (11.21)		5.8		2.21 (1.64-2.96)			<.001			457 (7.24)			607 (9.61)		2.37		1.36 (1.21-1.53)			<.001	
	Worry and anxiety	33 (2.55)		82 (6.34)		3.79		2.59 (1.71-3.9)			<.001			274 (4.34)			407 (6.44)		2.1		1.52 (1.3-1.78)			<.001		
	Low mood and not enjoying anything	57 (4.4)		97 (7.5)		3.1		1.76 (1.26-2.45)			.002			292 (4.62)			389 (6.16)		1.54		1.35 (1.16-1.58)			<.001		

^a^ONS: Office for National Statistics.

^b^OR: odds ratio.

^c^The *P* values by category of symptoms have had the Bonferroni correction applied for multiple testing.

^d^Inf: infinite.

### Comparison of People With COVID-19 Infection Without LC and Those With LC

The frequencies of baseline characteristics and univariate ORs for people with COVID-19 infection stratified by LC status are shown in Table 1 (n=416,505). The mean age was 44.5 (SD 21.77) years for the COVID-19 infection group and 47.7 (SD 14.8) years for the LC group. A higher proportion of those with LC was found among women (4926/7623, 64.62%), and male sex was associated with a lower odds of an LC diagnosis (OR 0.69, 95% CI 0.66-0.72). The proportion of those with a record of intensive care unit (ICU) admission was 0.63% (2580/408,882) in people with COVID-19 infection and 3.57% (272/7623) in people with LC, and a record of ICU admission was associated with a higher odds of an LC diagnosis (OR 5.83, 95% CI 5.13-6.62). A moderate association with LC was found for history of depression, anxiety, living in a conurbation, and COVID-19 vaccination at any time. A lower association was found for people with obesity, asthma, and hypertension.

**Figure 3:**
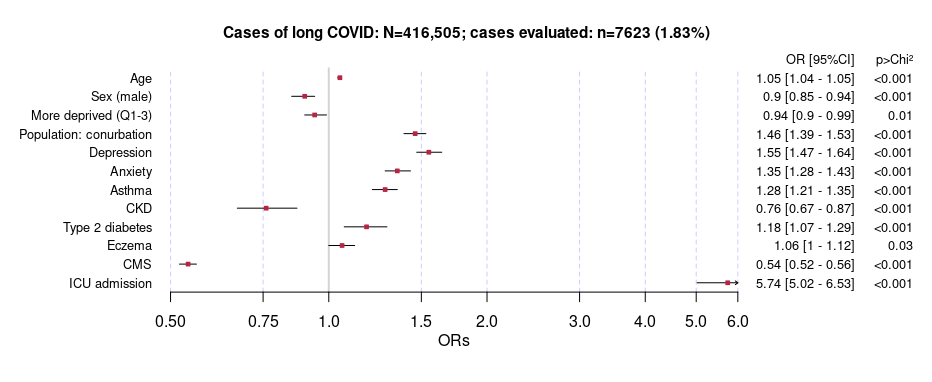
Multivariate logistic regression analysis showing associations with long COVID among people with COVID-19 infection diagnosed in the primary care sentinel cohort in England (March 1, 2020, to April 1, 2021). Results are shown as odds ratios (ORs) with 95% CIs. CKD: chronic kidney disease; CMS: Cambridge Multimorbidity Score; ICU: intensive care unit; Q: quintiles.

The multivariate logistic regression analysis using LC as an outcome (Figure 3) showed that a greater odds of having LC was associated with increasing age, higher population density (conurbation), mental health problems (anxiety and depression), and ICU admission. By contrast, male sex, being more deprived, chronic kidney disease (CKD), and a higher comorbidity score (measured using the CMS) were not.

An additional year of age was associated with a 5% increase in odds of an LC diagnosis (OR 1.05, 95% CI 1.04-1.05). After adjusting for confounders, the demographic factors associated with a decreased odds of an LC diagnosis among people with COVID-19 infection included male sex (OR 0.9, 95% CI 0.85-0.94) and higher deprivation (OR 0.94, 95% CI 0.9-0.99). However, residing in a conurbation was associated with increased odds of an LC diagnosis (OR 1.46, 95% CI 1.39-1.53). Among the history of comorbidities and exposures, depression (OR 1.55, 95% CI 1.47-1.64), anxiety (OR 1.35, 95% CI 1.28-1.35), asthma (OR 1.28, 95% CI 1.21-1.35), type 2 diabetes (OR 1.18, 95% CI 1.07-1.29), eczema (OR 1.06, 95% CI 1-1.12), and a record of ICU admission (OR 5.74, 95% CI 5.02-6.53) were associated with increased odds of an LC diagnosis. By contrast, history of CKD (OR 0.76, 95% CI 0.67-0.87) and a higher CMS (OR 0.54, 95% CI 0.52-0.56) were associated with lower odds of an LC diagnosis (Figure 3).

### Comparison of Posthospitalization- and Postcommunity Infection With LC

The frequencies of baseline characteristics and univariate ORs for people with LC stratified by community versus hospital index infection are shown in Table 3 (n=7623). Among baseline characteristics, the mean age was 54.8 (SD 14.3) years for the posthospitalization-infection LC group and 46.2 (SD 14.5) years for the postcommunity-infection LC group, whereas the proportion of female patients was 53.94% (705/1307) in the posthospitalization-infection LC group and 66.83% (4221/6316) in the postcommunity-infection LC group. Demographic factors associated with higher odds of hospitalization included male sex, higher deprivation, and non-White ethnicity. Among comorbidities, congestive heart failure, type 2 diabetes, CKD, ischemic heart disease, hypertension, chronic lung disease, obesity, and atrial fibrillation were all significantly associated with higher odds of a posthospitalization LC diagnosis.

**Figure 4:**
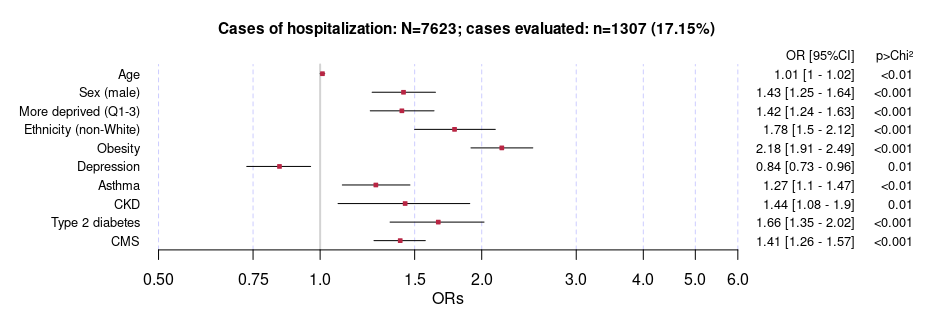
Multivariate logistic regression analysis showing associations with long COVID acquired after hospitalization. Results are shown as odds ratios (ORs) with 95% CIs. CKD: chronic kidney disease; CMS: Cambridge Multimorbidity Score; Q: quintiles.

The multivariate logistic regression analysis using posthospitalization LC as an outcome produced results (Figure 4) that contrasted with the previous analysis. Although increasing age, asthma, and type 2 diabetes remained associated with LC after both index community infection and hospitalization for the index COVID-19 infection, people who had received a diagnosis of LC after hospitalization were more likely to be men, more deprived, of non-White ethnicity, and have CKD and higher comorbidity scores (Figure 4).

An additional year of age was associated with a 1% increase in odds of having been hospitalized for COVID-19 infection (OR 1.01, 95% CI 1-1.02). After adjusting for confounders, the demographic factors associated with an increased odds of an LC diagnosis after hospitalization for COVID-19 infection included male sex (OR 1.43, 95% CI 1.25-1.64), higher deprivation (OR 1.42, 95% CI 1.24-1.63), non-White ethnicity (OR 1.78, 95% CI 1.5-2.12), and obesity (OR 2.18, 95% CI 1.9-2.5). Asthma (OR 1.27, 95% CI 1.1-1.47), CKD (OR 1.44, 95% CI 1.08-1.09), and type 2 diabetes (OR 1.66, 95% CI 1.35-2.02) were also associated with LC after hospitalization for COVID-19 infection. An increase in CMS was also associated with a 41% increase in odds of a posthospitalization LC diagnosis (OR 1.41, 95% CI 1.26-1.57). Only history of depression was associated with lower odds (OR 0.84, 95% CI 0.73-0.96).

**Table 3:** Frequencies of baseline characteristics and univariate odds ratios (ORs) for people with long COVID stratified by community versus hospital index infection in the primary care sentinel cohort in England (March 1, 2020, to April 1, 2021; N=7623).

Variable and category			Community, n=6316	Hospitalized, n=1307	Unadjusted odds ratio (95% CI)	*P* value
**Sociodemographic characteristics**						
	**Age (years), mean (SD)**					
		Continuous	46.2 (14.49)	54.8 (14.3)	1.04 (1.04-1.05)	<.001
	**Sex, n (%)**					
		Female (reference)	4221 (66.83)	705 (53.94)	1.00 (N/A^a^)	N/A
		Male	2095 (33.17)	602 (46.06)	1.72 (1.52-1.94)	<.001
	**Deprivation, n (%)**					
		Least deprived (reference)	2639 (41.78)	409 (31.29)	1.00 (N/A)	N/A
		Most deprived	3677 (58.22)	898 (68.71)	1.58 (1.39-1.79)	<.001
	**Ethnicity, n (%)**					
		White (reference)	4619 (73.13)	910 (69.63)	1.00 (N/A)	N/A
		Non-White	844 (13.36)	250 (19.13)	1.50 (1.28-1.76)	<.001
		Missing	853 (13.51)	147 (11.25)	0.87 (0.72-1.06)	.16
	**Population density, n (%)**					
		City (reference)	2639 (41.78)	552 (42.23)	1.00 (N/A)	N/A
		Conurbation	2621 (41.5)	575 (43.99)	1.05 (0.92-1.19)	.47
		Rural	1056 (16.72)	180 (13.77)	0.81 (0.68-0.98)	.03
	**BMI, n (%)**					
		Nonobese (reference)	3957 (62.65)	565 (43.23)	1.00 (N/A)	N/A
		Obese	1882 (29.8)	693 (53.02)	2.58 (2.28-2.92)	<.001
		Missing	477 (7.55)	49 (3.75)	0.72 (0.53-0.98)	.03
	**Smoker, n (%)**					
		Nonsmoker (reference)	3696 (58.52)	762 (58.3)	1.00 (N/A)	N/A
		Smoker or former smoker	2411 (38.17)	534 (40.86)	1.07 (0.95-1.21)	.25
		Missing	209 (3.31)	11 (0.84)	0.26 (0.14-0.47)	<.001
**Comorbidities**						
	**Depression, n (%)**					
		No (reference)	4029 (63.79)	833 (63.73)	1.00 (N/A)	N/A
		Yes	2287 (36.21)	474 (36.27)	1.00 (0.89-1.13)	.97
	**Anxiety, n (%)**					
		No (reference)	4094 (64.82)	875 (66.95)	1.00 (N/A)	N/A
		Yes	2222 (35.18)	432 (33.05)	0.91 (0.80-1.03)	.14
	**Asthma, n (%)**					
		No (reference)	4869 (77.09)	952 (72.84)	1.00 (N/A)	N/A
		Yes	1447 (22.91)	355 (27.16)	1.25 (1.10-1.44)	<.001
	**Chronic lung disease, n (%)**					
		No (reference)	6185 (97.93)	1244 (95.18)	1.00 (N/A)	N/A
		Yes	131 (2.07)	63 (4.82)	2.39 (1.76-3.25)	<.001
	**COPD^b^, n (%)**					
		No (reference)	6218 (98.45)	1255 (96.02)	1.00 (N/A)	N/A
		Yes	98 (1.55)	52 (3.98)	2.63 (1.87-3.70)	<.001
	**Hypertension, n (%)**					
		No (reference)	5168 (81.82)	833 (63.73)	1.00 (N/A)	N/A
		Yes	1148 (18.18)	474 (36.27)	2.56 (2.25-2.92)	<.001
	**Ischemic heart disease, n (%)**					
		No (reference)	6102 (96.61)	1181 (90.36)	1.00 (N/A)	N/A
		Yes	214 (3.39)	126 (9.64)	3.04 (2.42-3.82)	<.001
	**Atrial fibrillation, n (%)**					
		No (reference)	6228 (98.61)	1262 (96.56)	1.00 (N/A)	N/A
		Yes	88 (1.39)	45 (3.44)	2.52 (1.75-3.63)	<.001
	**Congestive heart failure, n (%)**					
		No (reference)	6283 (99.48)	1275 (97.55)	1.00 (N/A)	N/A
		Yes	33 (0.52)	32 (2.45)	4.78 (2.93-7.80)	<.001
	**CKD^c^, n (%)**					
		No (reference)	6155 (97.45)	1195 (91.43)	1.00 (N/A)	N/A
		Yes	161 (2.55)	112 (8.57)	3.58 (2.79-4.60)	<.001
	**Type 2 diabetes, n (%)**					
		No (reference)	5982 (94.71)	1060 (81.1)	1.00 (N/A)	N/A
		Yes	334 (5.29)	247 (18.9)	4.17 (3.50-4.98)	<.001
	**Type 1 diabetes, n (%)**					
		No (reference)	6282 (99.46)	1299 (99.39)	1.00 (N/A)	N/A
		Yes	34 (0.54)	8 (0.61)	1.14 (0.53-2.46)	.75
	**Cirrhosis, n (%)**					
		No (reference)	6305 (99.83)	1302 (99.62)	1.00 (N/A)	N/A
		Yes	11 (0.17)	5 (0.38)	2.20 (0.76-6.35)	.17
	**Eczema, n (%)**					
		No (reference)	4871 (77.12)	1020 (78.04)	1.00 (N/A)	N/A
		Yes	1445 (22.88)	287 (21.96)	0.95 (0.82-1.09)	.47
	**CMS^d^, mean (SD)**					
		Continuous	0.16 (1.03)	0.95 (1.33)	1.75 (1.66-1.84)	<.001
**Exposures**						
	**ICU^e^ admission, n (%)**					
		No (reference)	6316 (100)	1035 (79.19)	1.00 (N/A)	N/A
		Yes	0 (0)	272 (20.81)	Inf^f^	<.001
	**Vaccination at any time, n (%)**					
		No vaccine (reference)	743 (11.76)	129 (9.87)	1.00 (N/A)	N/A
		One dose	326 (5.16)	45 (3.44)	0.80 (0.55-1.14)	.21
		Two doses	5247 (83.07)	1133 (86.69)	1.24 (1.02-1.52)	.03
	**Pre–long COVID vaccination, n (%)**					
		No vaccine (reference)	6108 (96.71)	1239 (94.8)	1.00 (N/A)	N/A
		One dose	199 (3.15)	67 (5.13)	1.66 (1.25-2.20)	<.001
		Two doses	9 (0.14)	1 (0.08)	0.55 (0.07-4.33)	.54
**Outcomes**						
	**All-cause mortality, n (%)**					
		No (reference)	6310 (99.91)	1290 (98.7)	1.00 (N/A)	N/A
		Yes	6 (0.09)	17 (1.3)	13.9 (5.5-35.2)	<.001

^a^N/A: not applicable.

^b^COPD: chronic obstructive pulmonary disease.

^c^CKD: chronic kidney disease.

^d^CMS: Cambridge Multimorbidity Score.

^e^ICU: intensive care unit.

^f^Inf: infinite.

### LC and All-Cause Mortality

The frequencies of baseline characteristics and univariate ORs for people with LC stratified by vital status are shown in Table 4 (n=7623). We paired data for people with LC who died (23/7623, 0.3%) and those who remained alive within the study period (7600/7623, 99.7%). Demographic factors associated with a higher risk of mortality include male sex (OR 4.19, 95% CI 1.72-10.21) and age, where the mean age was 75.7 (SD 8.23) years in the mortality group and 47.6 (SD 14.75) years in the living group. Every additional year of age was associated with a 10% increased risk of death (OR 1.15, 95% CI 1.11-1.19). Among comorbidities, all cardiovascular comorbidities were associated with a higher risk of mortality, particularly congestive heart failure (OR 26, 95% CI 8.60-78.74) and atrial fibrillation (OR 20.8, 95% CI 8.06-53.54). Pulmonary conditions, including chronic obstructive pulmonary disease (OR 14.3, 95% CI 5.23-38.99) and chronic lung disease (OR 13.9, 95% CI 5.43-35.69) were also associated with higher mortality. Among metabolic and inflammatory conditions, cirrhosis, type 1 diabetes, type 2 diabetes, and eczema were associated with all-cause mortality. Finally, ICU admission (OR 7.63, 95% CI 2.81-20.70) was associated with a higher risk of death.

**Figure 5:**
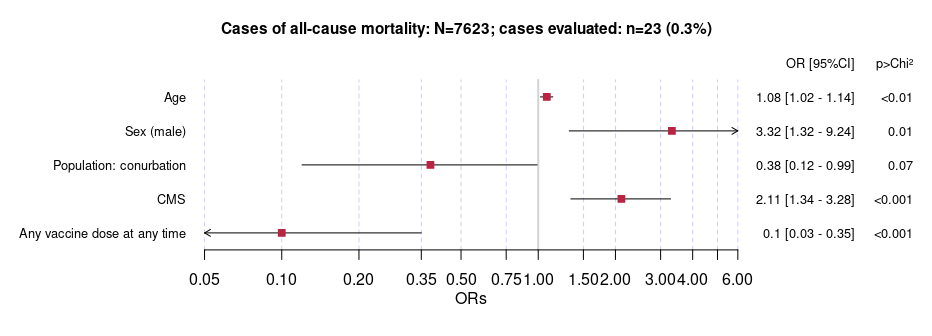
Multivariate logistic regression analysis of risk factors for all-cause mortality in people with long COVID. Results are shown as odds ratios (ORs) with 95% CIs. CMS: Cambridge Multimorbidity Score.

The results for the multivariate logistic regression analysis for people with LC using all-cause mortality as an outcome are presented in Figure 5. Age (OR 1.08, 95% CI 1.02-1.14), male sex, and a higher CMS were all associated with higher odds of mortality. By contrast, COVID-19 vaccination at any time and living in a conurbation were associated with lower odds of mortality (Figure 5).

**Table 4:** Frequencies of baseline characteristics and univariate odds ratios for people with LC stratified by vital status in the primary care sentinel cohort in England (March 1, 2020, to April 1, 2021; N=7623).

Variable and category						Alive, n=7600			All-cause mortality, n=23			Unadjusted odds ratio (95% CI)			*P* value
**Sociodemographic characteristics**															
	**Age (years), mean (SD)**														
		Continuous		47.6 (14.75)			75.7 (8.23)			1.15 (1.11-1.19)			<.001		
	**Sex, n (%)**														
		Female (reference)		4919 (64.72)			7 (30.43)			1.00 (N/A^a^)			N/A		
		Male		2681 (35.28)			16 (69.57)			4.19 (1.72-10.21)			<.001		
	**Deprivation, n (%)**														
		Least deprived (reference)		3042 (40.01)			6 (26.09)			1.00 (N/A)			N/A		
		Most deprived		4558 (59.97)			17 (73.9)			1.89 (0.74-4.80)			.16		
	**Ethnicity, n (%)**														
		White (reference)		5509 (72.49)			20 (86.96)			1.00 (N/A)			N/A		
		Non-White		1092 (14.37)			2 (8.7)			0.50 (0.12-2.16)			.31		
		Missing		999 (13.14)			1 (4.35)			0.28 (0.04-2.06)			.12		
	**Population density, n (%)**														
		City (reference)		3179 (41.83)			12 (52.17)			1.00 (N/A)			N/A		
		Conurbation		3191 (42)			5 (21.74)			0.42 (0.15-1.18)			.08		
		Rural		1230 (16.18)			6 (26.09)			1.29 (0.48-3.45)			.61		
	**BMI, n (%)**														
		Nonobese (reference)		4506 (59.29)			16 (69.57)			1.00 (N/A)			N/A		
		Obese		2568 (33.79)			7 (30.43)			0.77 (0.32-1.87)			.55		
		Missing		526 (6.92)			0 (0)			0.26 (0.02-4.33)			.06		
	**Smoker, n (%)**														
		Nonsmoker (reference)		4447 (58.51)			11 (47.82)			1.00 (N/A)			N/A		
		Smoker or former smoker		2933 (38.59)			12 (52.17)			1.65 (0.73- 3.75)			.23		
		Missing		220 (2.89)			0 (0)			0.88 (0.05-14.93)			.30		
**Comorbidities**															
	**Depression, n (%)**														
		No (reference)		4843 (63.72)			19 (82.61)			1.00 (N/A)			N/A		
		Yes		2757 (36.28)			4 (17.39)			0.37 (0.13-1.09)			.05		
	**Anxiety, n (%)**														
		No (reference)		4952 (65.16)			17 (73.91)			1.00 (N/A)			N/A		
		Yes		2648 (34.84)			6 (26.09)			0.66 (0.26-1.68)			.37		
	**Asthma, n (%)**														
		No (reference)		5802 (76.34)			19 (82.61)			1.00 (N/A)			N/A		
		Yes		1798 (23.66)			4 (17.39)			0.68 (0.23-2.00)			.47		
	**Chronic lung disease, n (%)**														
		No (reference)		7412 (97.53)			17 (73.91)			1.00 (N/A)			N/A		
		Yes		188 (2.47)			6 (26.09)			13.91 (5.43-35.69)			<.001		
	**COPD^b^ , n (%)**														
		No (reference)		7455 (98.09)			18 (78.26)			1.00 (N/A)			N/A		
		Yes		145 (1.91)			5 (21.74)			14.28 (5.23-38.99)			<.001		
	**Hypertension, n (%)**														
		No (reference)		5993 (78.86)			8 (34.78)			1.00 (N/A)			N/A		
		Yes		1607 (21.14)			15 (65.22)			6.99 (2.96-16.52)			<.001		
	**Ischemic heart disease, n (%)**														
		No (reference)		7265 (95.59)			18 (78.26)			1.00 (N/A)			N/A		
		Yes		335 (4.41)			5 (21.74)			6.02 (2.22-16.32)			<.001		
	**Atrial fibrillation, n (%)**														
		No (reference)		7473 (98.33)			17 (73.91)			1.00 (N/A)			N/A		
		Yes		127 (1.67)			6 (26.09)			20.77 (8.06-53.54)			<.001		
	**Congestive heart failure, n (%)**														
		No (reference)		7539 (99.2)			19 (82.61)			1.00 (N/A)			N/A		
		Yes		61 (0.8)			4 (17.39)			26.02 (8.60-78.74)			<.001		
	**CKD^c, n (%)^**														
		No (reference)		7332 (96.47)			18 (78.26)			1.00 (N/A)			N/A		
		Yes		268 (3.53)			5 (21.74)			7.60 (2.80-20.62)			<.001		
	**Type 2 diabetes, n (%)**														
		No (reference)		7028 (92.47)			14 (60.87)			1.00 (N/A)			N/A		
		Yes		572 (7.53)			9 (39.13)			7.90 (3.40-18.33)			<.001		
	**Type 1 diabetes, n (%)**														
		No (reference)		7559 (99.46)			22 (95.65)			1.00 (N/A)			N/A		
		Yes		41 (0.54)			1 (4.35)			8.38 (1.10-63.64)			.12		
	**Cirrhosis, n (%)**														
		No (reference)		7585 (99.8)			22 (95.65)			1.00 (N/A)			N/A		
		Yes		15 (0.2)			1 (4.35)			22.98 (2.91-181.62)			.04		
	**Eczema, n (%)**														
		No (reference)		5878 (77.34)			13 (56.52)			1.00 (N/A)			N/A		
		Yes		1722 (22.66)			10 (43.48)			2.63 (1.15-6.00)			.03		
	**CMS^d^, mean (SD)**														
		Continuous		0.28 (1.11)			3.37 (1.1)			3.24 (2.55-4.11)			<.001		
**Exposures**															
	**ICU^e^ admission, n (%)**														
		No (reference)		7333 (96.49)			18 (78.26)			1.00 (N/A)			N/A		
		Yes		267 (3.51)			5 (21.74)			7.63 (2.81-20.70)			<.001		
	**Vaccination at any time, n (%)**														
		No vaccine (reference)		867 (11.41)			5 (21.74)			1.00 (N/A)			N/A		
		One dose		367 (4.83)			4 (17.39)			1.89 (0.50-7.08)			.35		
		Two doses		6366 (83.76)			14 (60.87)			0.38 (0.14-1.06)			.09		
	**Pre–** **long COVID** **vaccination, n (%)**														
		No vaccine (reference)		7326 (96.39)			21 (91.3)			1.00 (N/A)			N/A		
		One dose		264 (3.47)			2 (8.7)			2.64 (0.62- 11.33)			.25		
		Two doses		10 (0.13)			0 (0)			Inf^f^			N/A		

^a^N/A: not applicable.

^b^COPD: chronic obstructive pulmonary disease.

^c^CKD: chronic kidney disease.

^d^CMS: Cambridge Multimorbidity Score.

^e^ICU: intensive care unit.

^f^Inf: infinite.

## Discussion

### Principal Findings

Although rates of recording of LC are low, an LC diagnosis was associated with an odds of more than twice as many consultations with ONS-defined LC-related symptoms in the 6 months after contracting the index infection compared with a historical control period. The increase in symptoms did not differ between those who had their initial COVID-19 infection managed in the hospital and those who were a community case. However, people with LC after hospitalization had greater odds of presenting with mental health problems, and those with LC after community infection had greater odds of presenting with general symptoms (weakness and tiredness, fever, myalgia, and abdominal pain).

There were some similarities, but there were marked contrasts between the characteristics of people who had been diagnosed with LC after a hospital infection and those who had been diagnosed with LC after a community infection. The similarities were associations with increasing age, asthma, and type 2 diabetes. The differences in the posthospitalization LC group were male sex, more deprivation, history of CKD, and higher multimorbidity scores, whereas those in the postcommunity LC group were female sex, less deprived, and more likely to have depression and anxiety as well as lower levels of comorbidities.

All-cause mortality in people with LC was higher in older men and those with higher multimorbidity scores, associations that were similar to those with hospitalized patients. COVID-19 vaccination was associated with lower odds of all-cause mortality in patients with LC.

### Comparison With the Literature

Posthospitalization LC was associated with higher deprivation, non-White ethnicity, obesity, CKD, and type 2 diabetes compared with postcommunity LC. There are similarities between our study, Living Risk Prediction Algorithm (QCOVID) study [21], and other studies [6,7] that reported the same risk factors, although with different outcomes. Data from the UK Health Security Agency and ONS indicated economic disadvantage to the prevalence of sex, obesity, diabetes, hypertension, and cardiovascular disease. These disparities may have been exacerbated because of the COVID-19 pandemic, especially among ethnic groups. However, there remains uncertainty regarding the degree to which the risk of developing posthospitalization LC in more deprived segments is linked to the severity of the disease (COVID-19) and more comorbidities [22] or to the propensity to consult in primary care, as reported for other respiratory conditions [23]. Disentangling the relationship between LC and hospital versus community is confounded by the presence of post-ICU syndrome in patients admitted to the ICU, many of the features of which (anxiety, cognitive difficulties, and breathlessness) overlap with LC. It is possible that the differences in the 2 groups are due to this confounding.

There are similarities between our study, Living Risk Prediction Algorithm (QCOVID) study [21], and other reports [3,24] regarding sociodemographic and comorbidity variables associated with mortality. These include cardiometabolic conditions (eg, CKD, type 2 diabetes, ischemic heart disease, and atrial fibrillation), chronic obstructive pulmonary disease, and asthma. However, we additionally report on vaccination status and conurbation as associated with lower odds of death. A UK Health Security Agency report indicated twice the risk of death due to COVID-19 infection among people from Bangladesh in comparison with White British people. Caribbean, Indian, Pakistani, Chinese, and Black ethnic groups were identified to have a 10% to 50% higher risk of mortality in comparison with White British people. We do not report differences on mortality per ethnicity because the number of deaths in the LC group was too small to enable us to find differences across groups.

We identified a lower prevalence of LC compared with self-report population surveys (eg, ONS) [4], but the prevalence was higher than those reported by other studies using routine data [9]. The OpenSAFELY study found that 27% of practices had no LC recording compared with 7.8% in our sentinel cohort [9]. Low rates of clinical coding of LC and interpractice variation are a national problem. COVID-19 coding evolved over the course of the pandemic, and the United Kingdom also has a region-specific version of SNOMED CT, which further complicates the issue [19,25]. LC clinical codes were introduced to SNOMED CT in January 2021; therefore, primary care staff did not have access to these until February 2021 [9,10]. Levels recorded in GP CMRs are dependent both on patients consulting their GP (many do not) and the GP both recognizing LC and coding it; therefore, our estimate of 1.83% (7623/416,505) will be very conservative. In addition, vaccination has been shown to decrease the risk of developing LC by approximately half; therefore, risks will have fallen over time (ONS).

### Strengths and Limitations

The data were sourced from a representative network (PCSC) where practices have received feedback throughout the pandemic. Data on COVID-19 infection diagnoses and comorbidity are likely to be of good quality [17,26]. Linkage to hospital and mortality data adds reliability compared with only using coded data. Clinicians may well be underrecording LC, either by not recognizing it or by coding it with a *presenting symptom* code. The ONS symptoms are the most well-validated set of LC symptoms available at present; however, a validation study is pending. In addition, LC may be diagnosed as other conditions and coded as such; for example, a patient with pre-existing anxiety may well have their LC-related tachycardia and breathlessness diagnosed as worsening anxiety. It is known that GPs tend not to record symptoms reliably in the record and tend to only record symptoms that support their working diagnosis, introducing bias [27]. As a result, vital data may not be coded and included in this study because of these being available as free text within CMRs. Considerable care needs to be taken in interpreting these very granular data from the CMR.

### Implications of the Findings

It is very likely that LC was underrecorded, and clinicians should continue to be encouraged to record this condition in clinical records. LC is an important comorbidity that needs to be captured so that it can be taken into consideration in health service planning and assessment of vaccine benefit risk.

The differences between those presenting to community and hospital care may have represented the propensity of different groups to consult (eg, women more than men in primary care) and the possibility that some groups experienced more serious disease (eg, men and increasing age), as well as the potential for disparities in presentation.

As COVID-19 continues to circulate in the community, albeit with lower death and morbidity rates because of vaccines and a current less-virulent strain, learning to recognize groups of patients at greatest risk of acquiring LC and managing associated risk factors may affect presentations and disease impact. Comorbid conditions that increase the risk of acquiring LC, such as asthma, may shed light on possible etiological risk factors.

### Call for Further Research

LC case identification within primary care requires improved data recording. Better case identification will enable successful interventions to be implemented. A range of incentives to improve case ascertainment and data quality have been successfully implemented in primary care. This would further aid in understanding communicable disease risk and to develop better controls in the future.

### Conclusions

LC recording in primary care records was low, reflecting either low prevalence or underrecording. There are differences between the sociodemographic profiles and comorbidities of LC symptoms presented after an index community infection compared with those hospitalized with a COVID-19 infection. Factors associated with hospital presentation are also associated with higher all-cause mortality, although vaccination is protective. This suggests that the disparities flagged throughout the COVID-19 pandemic may also apply to LC, where better tools to identify and intervene are needed in those at greatest risk.

## Abbreviations

CKD: chronic kidney disease
CMR: computerized medical record
CMS: Cambridge Multimorbidity Score
GP: general practitioner
ICU: intensive care unit
ONS: Office for National Statistics
OR: odds ratio
PCSC: primary care sentinel cohort
SNOMED CT: Systematized Nomenclature of Medicine Clinical Terms

## References

[1] Sivan M, Rayner C, Delaney B (2021). Fresh evidence of the scale and scope of long covid. BMJ.

[2] Soriano JB, Murthy S, Marshall JC, Relan P, Diaz JV (2022). A clinical case definition of post-COVID-19 condition by a Delphi consensus. Lancet Infect Dis.

[3] Ayoubkhani D, Khunti K, Nafilyan V, Maddox T, Humberstone B, Diamond I, Banerjee A (2021). Post-covid syndrome in individuals admitted to hospital with covid-19: retrospective cohort study. BMJ.

[4] Prevalence of ongoing symptoms following coronavirus (COVID-19) infection in the UK : 3 February 2022. Office of National Statistics.

[5] Davis HE, Assaf GS, McCorkell L, Wei H, Low RJ, Re'em Y, Redfield S, Austin JP, Akrami A (2021). Characterizing long COVID in an international cohort: 7 months of symptoms and their impact. EClinicalMedicine.

[6] Sudre CH, Murray B, Varsavsky T, Graham MS, Penfold RS, Bowyer RC, Pujol JC, Klaser K, Antonelli M, Canas LS, Molteni E, Modat M, Jorge Cardoso M, May A, Ganesh S, Davies R, Nguyen LH, Drew DA, Astley CM, Joshi AD, Merino J, Tsereteli N, Fall T, Gomez MF, Duncan EL, Menni C, Williams FM, Franks PW, Chan AT, Wolf J, Ourselin S, Spector T, Steves CJ (2021). Attributes and predictors of long COVID. Nat Med.

[7] Aiyegbusi OL, Hughes SE, Turner G, Rivera SC, McMullan C, Chandan JS, Haroon S, Price G, Davies EH, Nirantharakumar K, Sapey E, Calvert MJ, Study Group TL (2021). Symptoms, complications and management of long COVID: a review. J R Soc Med.

[8] Deer RR, Rock MA, Vasilevsky N, Carmody L, Rando H, Anzalone AJ, Basson MD, Bennett TD, Bergquist T, Boudreau EA, Bramante CT, Byrd JB, Callahan TJ, Chan LE, Chu H, Chute CG, Coleman BD, Davis HE, Gagnier J, Greene CS, Hillegass WB, Kavuluru R, Kimble WD, Koraishy FM, Köhler S, Liang C, Liu F, Liu H, Madhira V, Madlock-Brown CR, Matentzoglu N, Mazzotti DR, McMurry JA, McNair DS, Moffitt RA, Monteith TS, Parker AM, Perry MA, Pfaff E, Reese JT, Saltz J, Schuff RA, Solomonides AE, Solway J, Spratt H, Stein GS, Sule AA, Topaloglu U, Vavougios GD, Wang L, Haendel MA, Robinson PN (2021). Characterizing long COVID: deep phenotype of a complex condition. EBioMedicine.

[9] Walker AJ, MacKenna B, Inglesby P, Tomlinson L, Rentsch CT, Curtis HJ, Morton CE, Morley J, Mehrkar A, Bacon S, Hickman G, Bates C, Croker R, Evans D, Ward T, Cockburn J, Davy S, Bhaskaran K, Schultze A, Williamson EJ, Hulme WJ, McDonald HI, Mathur R, Eggo RM, Wing K, Wong AY, Forbes H, Tazare J, Parry J, Hester F, Harper S, O'Hanlon S, Eavis A, Jarvis R, Avramov D, Griffiths P, Fowles A, Parkes N, Douglas IJ, Evans SJ, (The OpenSAFELY Collaborative) (2021). Clinical coding of long COVID in English primary care: a federated analysis of 58 million patient records using OpenSAFELY. Br J Gen Pract.

[10] Mayor N, Tsang R, Joy M, Hobbs FR, de Lusignan S (2021). Long covid: coding is caring. BMJ.

[11] de Lusignan S, Dorward J, Correa A, Jones N, Akinyemi O, Amirthalingam G, Andrews N, Byford R, Dabrera G, Elliot A, Ellis J, Ferreira F, Lopez Bernal J, Okusi C, Ramsay M, Sherlock J, Smith G, Williams J, Howsam G, Zambon M, Joy M, Hobbs FD (2020). Risk factors for SARS-CoV-2 among patients in the Oxford Royal College of General Practitioners Research and Surveillance Centre primary care network: a cross-sectional study. Lancet Infect Dis.

[12] de Lusignan S, Joy M, Oke J, McGagh D, Nicholson B, Sheppard J, Akinyemi O, Amirthalingam G, Brown K, Byford R, Dabrera G, Krajenbrink E, Liyanage H, LopezBernal J, Okusi C, Ramsay M, Sherlock J, Sinnathamby M, Tsang RS, Tzortziou Brown V, Williams J, Zambon M, Ferreira F, Howsam G, Hobbs FR (2020). Disparities in the excess risk of mortality in the first wave of COVID-19: cross sectional study of the English sentinel network. J Infect.

[13] Espinosa-Gonzalez AB, Neves AL, Fiorentino F, Prociuk D, Husain L, Ramtale SC, Mi E, Mi E, Macartney J, Anand SN, Sherlock J, Saravanakumar K, Mayer E, de Lusignan S, Greenhalgh T, Delaney BC (2021). Predicting risk of hospital admission in patients with suspected COVID-19 in a community setting: protocol for development and validation of a multivariate risk prediction tool. JMIR Res Protoc.

[14] Mayer N, Meza-torres B, Okusi C, Delanerolle G, Wang W, Anand S (2022). Developing a long covid phenotype for post-acute COVID-19 in a national primary care sentinel cohort: an observational retrospective database analysis. JMIR Preprints.

[15] Puntmann VO, Carerj ML, Wieters I, Fahim M, Arendt C, Hoffmann J, Shchendrygina A, Escher F, Vasa-Nicotera M, Zeiher AM, Vehreschild M, Nagel E (2020). Outcomes of cardiovascular magnetic resonance imaging in patients recently recovered from coronavirus disease 2019 (COVID-19). JAMA Cardiol.

[16] Joy M, Hobbs FR, Bernal JL, Sherlock J, Amirthalingam G, McGagh D, Akinyemi O, Byford R, Dabrera G, Dorward J, Ellis J, Ferreira F, Jones N, Oke J, Okusi C, Nicholson BD, Ramsay M, Sheppard JP, Sinnathamby M, Zambon M, Howsam G, Williams J, de Lusignan S (2020). Excess mortality in the first COVID pandemic peak: cross-sectional analyses of the impact of age, sex, ethnicity, household size, and long-term conditions in people of known SARS-CoV-2 status in England. Br J Gen Pract.

[17] de Lusignan S (2005). Codes, classifications, terminologies and nomenclatures: definition, development and application in practice. Inform Prim Care.

[18] Payne RA, Mendonca SC, Elliott MN, Saunders CL, Edwards DA, Marshall M, Roland M (2020). Development and validation of the Cambridge Multimorbidity Score. CMAJ.

[19] de Lusignan S, Liyanage H, McGagh D, Jani BD, Bauwens J, Byford R, Evans D, Fahey T, Greenhalgh T, Jones N, Mair FS, Okusi C, Parimalanathan V, Pell JP, Sherlock J, Tamburis O, Tripathy M, Ferreira F, Williams J, Hobbs FD (2020). COVID-19 surveillance in a primary care sentinel network: in-pandemic development of an application ontology. JMIR Public Health Surveill.

[20] Data Security and Protection Toolkit. NHS Digital.

[21] Clift AK, Coupland CA, Keogh RH, Diaz-Ordaz K, Williamson E, Harrison EM, Hayward A, Hemingway H, Horby P, Mehta N, Benger J, Khunti K, Spiegelhalter D, Sheikh A, Valabhji J, Lyons RA, Robson J, Semple MG, Kee F, Johnson P, Jebb S, Williams T, Hippisley-Cox J (2020). Living risk prediction algorithm (QCOVID) for risk of hospital admission and mortality from coronavirus 19 in adults: national derivation and validation cohort study. BMJ.

[22] de Lusignan S, McGee C, Webb R, Joy M, Byford R, Yonova I, Hriskova M, Matos Ferreira F, Elliot AJ, Smith G, Rafi I (2018). Conurbation, urban, and rural living as determinants of allergies and infectious diseases: royal college of general practitioners research and surveillance centre annual report 2016-2017. JMIR Public Health Surveill.

[23] de Lusignan S, Correa A, Pathirannehelage S, Byford R, Yonova I, Elliot AJ, Lamagni T, Amirthalingam G, Pebody R, Smith G, Jones S, Rafi I (2016). RCGP Research and Surveillance Centre Annual Report 2014–2015: disparities in presentations to primary care. Br J Gen Pract.

[24] Pérez-González A, Araújo-Ameijeiras A, Fernández-Villar A, Crespo M, Poveda E, Cabrera J (2022). Long COVID in hospitalized and non-hospitalized patients in a large cohort in Northwest Spain, a prospective cohort study. Sci Rep.

[25] de Lusignan S, Williams J (2020). To monitor the COVID-19 pandemic we need better quality primary care data. BJGP Open.

[26] de Lusignan S, Correa A, Smith GE, Yonova I, Pebody R, Ferreira F, Elliot AJ, Fleming D (2017). RCGP Research and Surveillance Centre: 50 years’ surveillance of influenza, infections, and respiratory conditions. Br J Gen Pract.

[27] Kostopoulou O, Tracey C, Delaney B (2021). Can decision support combat incompleteness and bias in routine primary care data?. J Am Med Inform Assoc.

